# Development and validation of a house finch interleukin-1β (HfIL-1β) ELISA system

**DOI:** 10.1186/s12917-017-1199-9

**Published:** 2017-08-30

**Authors:** Sungwon Kim, Myeongseon Park, Ariel E. Leon, James S. Adelman, Dana M. Hawley, Rami A. Dalloul

**Affiliations:** 1 Avian Immunobiology Laboratory, Department of Animal and Poultry Sciences, Virginia Tech, Blacksburg, VA 24061 USA; 2 The Roslin Institute and R(D)SVS, University of Edinburgh, Easter Bush, Midlothian, EH25 9RG UK; 3 Department of Biological Sciences, Virginia Tech, Blacksburg, VA 24061 USA; 4 Department of Natural Resource Ecology and Management, Iowa State University, Ames, IA 50011 USA

**Keywords:** Interleukin-1beta, ELISA, House finch, *Mycoplasma gallisepticum*

## Abstract

**Background:**

A unique clade of the bacterium *Mycoplasma gallisepticum* (MG), which causes chronic respiratory disease in poultry, has resulted in annual epidemics of conjunctivitis in North American house finches since the 1990s. Currently, few immunological tools have been validated for this songbird species. Interleukin-1β (IL-1β) is a prototypic multifunctional cytokine and can affect almost every cell type during *Mycoplasma* infection. The overall goal of this study was to develop and validate a direct ELISA assay for house finch IL-1β (HfIL-1β) using a cross-reactive chicken antibody.

**Methods:**

A direct ELISA approach was used to develop this system using two different coating methods, carbonate and dehydration. In both methods, antigens (recombinant HfIL-1b or house finch plasma) were serially diluted in carbonate-bicarbonate coating buffer and either incubated at 4 °C overnight or at 60 °C on a heating block for 2 hr. To generate the standard curve, rHfIL-1b protein was serially diluted at 0, 3, 6, 9, 12, 15, 18, 21, and 24 ng/mL. Following blocking and washing, anti-chicken IL-1b polyclonal antibody was added, plates were later incubated with detecting antibodies, and reactions developed with tetramethylbenzidine solution.

**Results:**

A commercially available anti-chicken IL-1β (ChIL-1β) polyclonal antibody (pAb) cross-reacted with house finch plasma IL-1β as well as bacterially expressed recombinant house finch IL-1β (rHfIL-1β) in immunoblotting assays. In a direct ELISA system, rHfIL-1β could not be detected by an anti-ChIL-1β pAb when the antigen was coated with carbonate-bicarbonate buffer at 4°C overnight. However, rHfIL-1β was detected by the anti-ChIL-1β pAb when the antigen was coated using a dehydration method by heat (60°C). Using the developed direct ELISA for HfIL-1β with commercial anti-ChIL-1β pAb, we were able to measure plasma IL-1β levels from house finches.

**Conclusions:**

Based on high amino acid sequence homology, we hypothesized and demonstrated cross-reactivity of anti-ChIL-1β pAb and HfIL-1β. Then, we developed and validated a direct ELISA system for HfIL-1β using a commercial anti-ChIL-1β pAb by measuring plasma HfIL-1β in house finches.

## Background

A member of the interleukin (IL)-1 family, IL-1beta (IL-1β) is a pivotal pro-inflammatory cytokine for host-defense responses to infection and injury [1–3]. It is a major mediator of innate immunity, as well as adaptive immune responses. The guardian cells of the innate immune system – macrophages and monocytes – are a major source of IL-1β [4, 5], but many other cell types including epithelial cells [6], endothelial cells [7], and fibroblasts [8] can also produce this cytokine. IL-1β is produced as an inactive 31 kDa precursor – termed pro-IL-1β, which is proteolytically processed to its active form by cytosolic caspase-1, followed by secretion via an unconventional protein secretion pathway [9]. Activated IL-1β affects diverse major innate immune processes including immune cell recruitment, cell proliferation, tissue destruction, bone resorption, vascular smooth muscle cell contraction, blood pressure and central nervous system cell function [10, 11].

A chicken homolog of mammalian IL-1β was first identified and characterized with CXCLi1 (K60)-inducing activity in 1998 [12], and some research has focused on its biological roles in avian species. Expression of ChIL-1β typically increases in response to both bacterial and viral infections, consistent with its role as a rapidly induced pro-inflammatory mediator. For example, IL-1β expression in bursal cells increases in chickens infected with infectious bursal disease virus [13], and IFN-γ-primed heterophils stimulated with *Salmonella* Enteritidis show increased IL-1β expression [14]. Infection of chicken kidney cells (CKCs) with *Escherichia coli* caused a reduction of IL-1β compared to non-infected control, but infection with *S*. *typhimurium* or *S*. *dublin* led to significantly increased IL-1β transcripts [15]. Chicken macrophage cell line (HD11) and CKCs induced significant IL-1β mRNA expression during stimulation with *Campylobacter jejuni* [16]. Finally, a study of *Mycoplasma gallisepticum* (MG) infection in chickens revealed the down-regulation of IL-1β at 1-day post-inoculation (dpi), and then a three-fold increase in expression at 4-dpi [17].

The bacterium MG is a common cause of chronic respiratory disease of poultry, but a unique clade of this pathogen emerged in a common North American backyard songbird species, house finch (*Haemorhous mexicanus*), in the mid-1990s [18, 19]. MG causes severe conjunctivitis in finches and significantly reduces survival in free-living birds [20, 21]. This pathogen spreads by either direct contact or short-term indirect contact on bird feeders, and MG is now endemic throughout most of the house finch range in North America [21, 22]. MG in house finches induces a series of local and systemic inflammatory responses, including severe conjunctivitis and rhinitis [21], local infiltration of lymphocytes and heterophils [23], and systemic responses such as fever, sickness behaviors, and expression of pro-inflammatory cytokines [24, 25]. A recent microarray study reported a different gene expression profile during innate and adaptive immune responses between MG-resistant (Alabama population) and MG-susceptible (Arizona population) finches [26]. MG-susceptible finches exhibited significant down-regulation of gene expression patterns at 3-dpi (innate response) and 14-dpi (adaptive response) compared to MG-resistant finches. These two populations showed distinct transcriptional responses in the early stages of infection. Additionally, while gene expression profiles were similar on 3- and 14-dpi in MG-susceptible finches, MG-resistant finches had significantly different gene expression profile between 3-dpi and 14-dpi, suggesting genes associated with the adaptive immune response are only up-regulated after population differences in transcription are first observed [26]. We recently documented population differences in relative IL-1β mRNA expression early in experimental MG infection, and these expression differences correspond to population differences in the severity of conjunctivitis [25]. However, a full understanding of the role of IL-1β in MG infection requires tools to measure and potentially manipulate the IL-1β protein in house finches.

Previously, our team identified and characterized HfIL-1β including successfully expressing rHfIL-1β in both prokaryotic and eukaryotic systems [27]. Recombinant HfIL-1β induced cell proliferation, as well as nitric oxide production in house finch splenocytes. Additionally, rHfIL-1β resulted in increased mRNA levels of Th1/Th2 cytokines in splenocytes, as well as an acute phase protein and an antimicrobial peptide in hepatocytes [27]. The goal of this study was to develop and validate a direct ELISA system for house finch IL-1β (HfIL-1β) using a commercially available anti-chicken IL-1β (ChIL-1β). Based on the high amino acid sequence homology of IL-1β between chicken and house finch, our previous work demonstrated the cross-reactivity of anti-ChIL-1β polyclonal antibody (pAb) to recombinant HfIL-1β [27]. In this study, we validated the cross-reactivity of anti-ChIL-1β pAb to nature form of serum HfIL-1β by immunoblotting. Then, a direct ELISA system using anti-ChIL-1β pAb was developed and test-validated with plasma samples collected from house finches.

## Methods

### Blood samples

Blood was collected from house finches via wing vein puncture using heparinized microcapillary blood collection tubes (approximately 100 μL per bird) and plasma was separated via centrifugation and frozen at −20°C. Once thawed, all blood samples were further diluted with PBS for the immunoblotting and ELISA analyses. Plasma samples from four captive house finches (non-infected with MG) were randomly selected for assay development (Table 1). Plasma samples from four free-living house finches were used to test whether HfIL-1β levels are elevated during MG infection: two of these individuals were clinically healthy at capture and were thus considered non-infected, and the other individuals had severe clinical signs of mycoplasmal conjunctivitis and thus were considered MG-infected. All housing and animal procedures were approved by the Institutional Animal Care and Use Committee of Virginia Tech.

**Table 1:** Bird identification for blood collection

Bird ID	Infection	Capture Date	Status
380	No	07/13/2012	Had been captive for >1 year, but always control bird (non-infected)
412	No	16/01/2012	
1401	Yes	24/07/2013	Captured in the field without pathology, broke with MG while housed in captivity prior to time of sampling
1410	Yes	26/07/2013	

### Immunoblotting

Concentration of the purified recombinant HfIL-1β (rHfIL-1β) was measured using a BCA Protein Assay Kit (Pierce, IL) following the manufacturer’s instructions. For SDS-PAGE, 1 μg of the purified protein or 1 μL of plasma samples was mixed with 10 μL of SDS loading buffer (New England Biolabs, MA) containing DTT. The samples were then boiled at 97°C on a hot plate for 7 min. The prepared samples were electrophoresed on a 12% SDS-polyacrylamide gel at 90 V for 140 min. Proteins were transferred to PVDF membranes (Millipore, MA) by the submarine method at 90 V for 1.5 h. The membranes were incubated with anti-ChIL-1β pAb (Thermo Scientific, IL; 1:1000) overnight, followed by incubation with goat anti-rabbit antibody conjugated with HRP (Santa Cruz Biotechnology, CA, 1:2000) for 45 min. Using the SuperSignal West Pico Chemiluminescent Substrate (Pierce, IL), HRP signal was enhanced, followed by exposure and development on CL-XPosure film (Thermo Scientific, IL).

### Enzyme-linked Immunosorbent assay (ELISA)

To develop the ELISA system for HfIL-1β, we adopted a direct ELISA approach using two different coating methods, carbonate and dehydration, on Nunc MaxiSorp® flat-bottom 96-well plates (Thermo Scientific, IL). In both methods, antigens – either rHfIL-1β or plasma – were diluted in carbonate-bicarbonate coating buffer (0.05 M; pH 9.5). For the standard curve, rHfIL-1β protein was serially diluted with 0.05 M carbonate-bicarbonate buffer at the following concentrations: 0, 3, 6, 9, 12, 15, 18, 21, and 24 ng/mL. The plasma samples were diluted with carbonate-bicarbonate coating buffer (0.05 M; pH 9.5) using 10-fold dilutions. An aliquot of 100 μL of each diluted antigen was added to the assigned well of the 96-well plates. For the carbonate method, the plates were then incubated at 4°C overnight, while for the dehydration method, the plates were incubated at 60°C on a heating block for 2 h for complete dehydration. Then, each plate was incubated with a blocking buffer (PBS [pH 7.4] containing 0.05% Tween-20 and 1% BSA) at room temperature (RT) for 45 min.

The primary (rabbit anti-ChIL-1β pAb) and the secondary (goat anti-rabbit) antibodies were prepared by dilution with the blocking buffer at 1:1000 and 1:2000, respectively. Fifty microliters of anti-ChIL-1β pAb were added to each assigned well, followed by incubating on a microplate shaker for 1 h at RT and washing 3 times (washing buffer as PBS [pH 7.4] containing 0.05% Tween-20). The plates were then incubated with 50 μL of the goat anti-rabbit antibody for 1 h at RT with continuous shaking. To develop HRP signal, a 3.3′,5.5′-tetramethylbenzidine (TMB, Sigma-Aldrich, MO) solution was prepared in 1 mL DMSO, 9 mL of 0.05 M phosphate-citrate buffer (pH 5.0) and 2 μL of 30% hydrogen peroxide (0.03% as final concentration, Sigma-Aldrich) per 10 mL total volume. One hundred microliters of the prepared TMB solution were added to each well, followed by incubation for 30–45 min at RT. Colorimetric development was stopped by adding 100 μL of 2 N sulfuric acid (H_2_SO_4_) then absorbance was quantified spectrophotometrically at 450 nm with a microplate reader. The raw OD values were normalized by subtracting OD value of buffer. The standard curve was drawn and calculated with Excel (Microsoft Corp., WA) and elisaanalysis.com (www.elisaanalysis.com) using a logarithmic equation (i.e. Y = aln (x) + b), which was applied to calculate the concentration of HfIL-1β. The data were analyzed by independent two-sample *t*-test using program R (https://www.r-project.org/).

## Results

### Cross-reactivity of commercial anti-ChIL-1β antibody to HfIL-1β

**Fig. 1:**
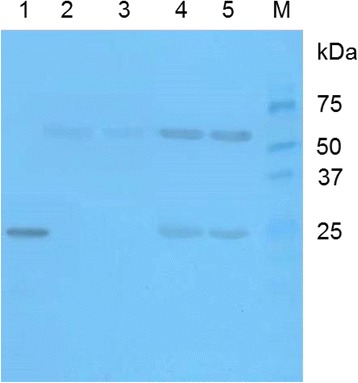
Cross-reactivity of anti-ChIL-1β antibody wiht HfIL-1β. One-microgram of rHfIL-1β expressed from *E. coli* (lane 1), 1 μL of plasma from non-infected (lanes 2, 3) and MG-infected birds (lanes 4, 5) were analyzed by immunoblotting using anti-ChIL-1β antibody. M represents protein molecular weight marker (kDa)

To validate cross-reactivity of commercially available anti-ChIL-1β pAb with HfIL-1β, immunoblotting was performed with rHfIL-1β (Fig. 1); we also included plasma samples from non-infected and MG-infected house finches to verify such cross-reactivity. Approximately 25 kDa of rHfIL-1β was clearly detected by anti-ChIL-1β pAb. Plasma from non-infected birds showed only approximately 60 kDa protein band, whereas MG-infected birds showed 25 kDa and 60 kDa protein bands. The immunoblot results verified that commercial anti-ChIL-1β pAb can cross-react with both recombinant and natural forms of HfIL-1β.

### Development of direct ELISA assay using anti-ChIL-1β antibody

**Fig. 2:**
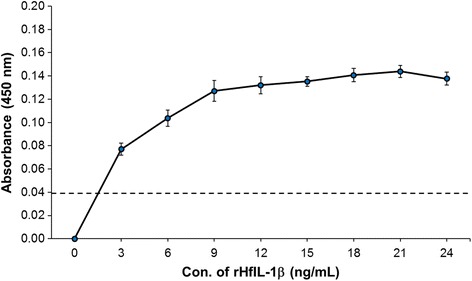
A standard curve with rHfIL-1β by dehydration coating method. To establish an ELISA system for HfIL-1β, first a standard curve was established with rHfIL-1β using the dehydration coating method. The purified rHfIL-1β was diluted with the coating buffer with the following final concentrations: 0, 3, 6, 9, 12, 15, 15, 18, 21, and 24 ng/mL. Fifty-microliter of serially diluted rHfIL-1β were added to each well of Nunc MaxiSorp® flat-bottom 96-well plate, followed by incubation at 60°C for 2 h. Then, the plate was sequentially incubated with anti-ChIL-1β pAb (1:1000) and goat anti-rabbit antibody (1: 2000). The HRP signal was developed with TMB solution for 30 min. The coating buffer itself was used as negative control. Values represent the mean of three independent experiments. Error bars represent standard error of the mean. The dashed line indicates the threshold line, representing the value of negative control and limitation of the developed ELISA system (OD450 = 0.038)

To develop a direct ELISA system for HfIL-1β, we first tested the carbonate method for antigen coating. However, there was no signal detected even when coating with 2000 ng/mL rHfIL-1β (data not shown). To improve coating the antigen on the plate, the dehydration method was adopted. The ELISA results showed increased optical density (OD450) in a dose-dependent fashion (Fig. 2). The negative controls (coating buffer and the goat anti-rabbit antibody) were only in the 0.034–0.044 range. Using the dehydration coating method, we were able to detect as low as 2 ng/mL of rHfIL-1β protein. The standard curve showed a logarithmic scale (Y = a – b*ln (x + c)) with 0.94–0.95 adjusted R-square value.

### Validation of the developed ELISA system using house finch plasma

**Fig. 3:**
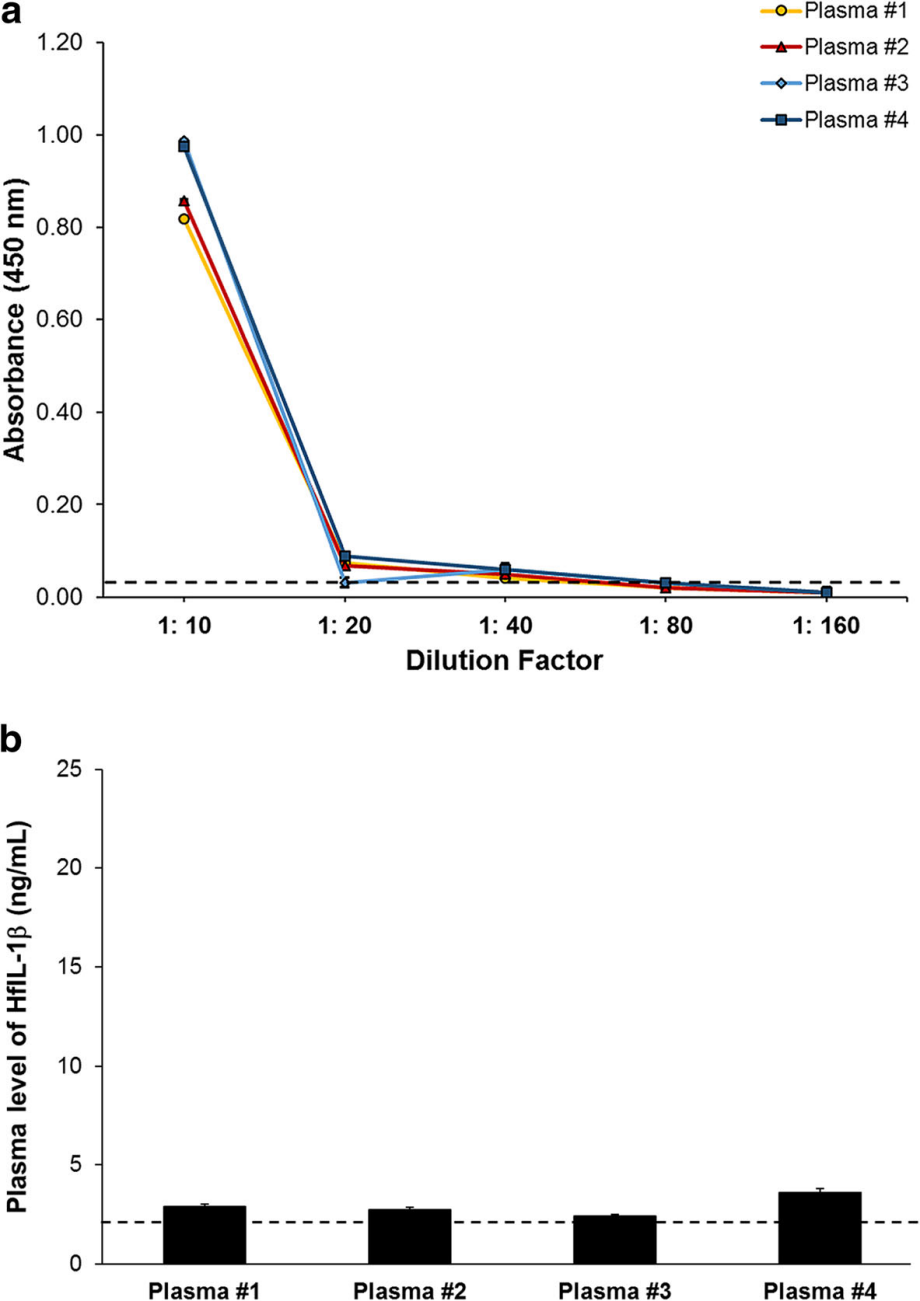
Quantification of HfIL-1β in the plasma of non-infected house finches using the developed ELISA system. House finch plasma from randomly-selected non-infected birds were serially diluted with PBS as follows: 1:10, 1:20, 1:40, 1:80, and 1:160 (**a**). The diluted samples were coated with the dehydration method (60°C for 2 h), followed by sequential incubation with anti-ChIL-1β antibody (1:1000) as the primary antibody and the secondary antibody goat anti-rabbit antibody (1:2000). The HRP signal was developed with TMB solution for 45 min. The concentration of HfIL-1β plasma levels was calculated using Excel (Microsoft Corp) (**b**). The values represent the mean of triplicate wells with standard deviation bars. The dashed line indicates the threshold line, representing the value of negative control and limitation of the developed ELISA system (OD450 = 0.034)

To validate the developed ELISA system, we measured HfIL-1β plasma level from randomly selected captive house finches. The samples were diluted with coating buffer at 1:10 to 1:160 dilution factors (Fig. 3a), followed by coating using the dehydration method. Based on the OD450 values, the plasma samples diluted at 1:20 showed a similar range as the standard curve. Therefore, we used a 1:20 dilution of plasma samples to measure circulating HfIL-1β levels in house finches. The standard curve showed “Y = a - b*ln (x + c)” equation with: a = 0.08494 (± 0.01225), b = −0.01948 (± 0.00456), and c = −2.35041 (± 0.52948). This translates into 2.92 ng/mL, 2.77 ng/mL, 2.41 ng/mL and 3.58 ng/mL of plasma HfIL-1β in each house finch, respectively (Fig. 3b).

**Fig. 4:**
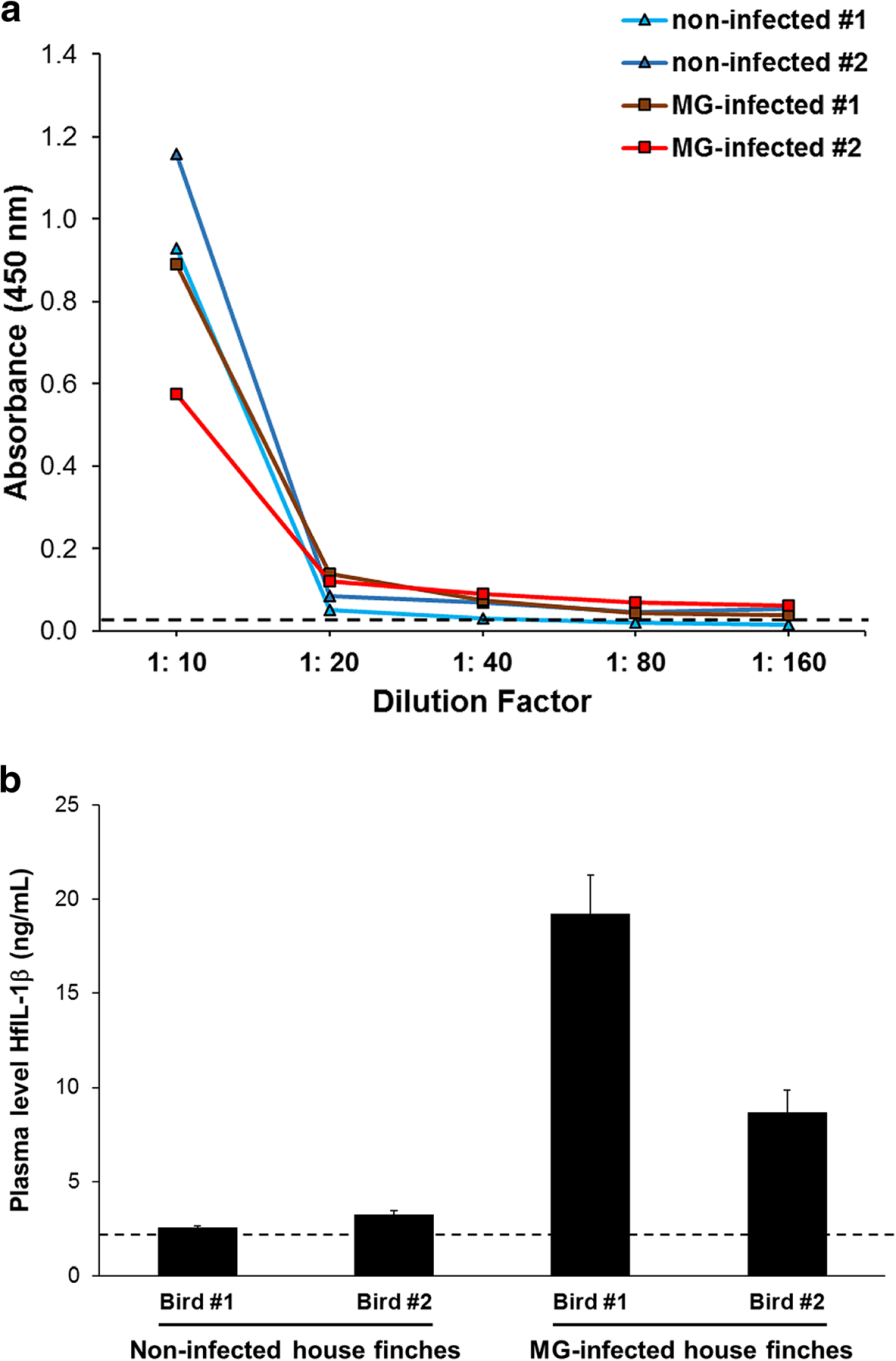
Quantification of plasma HfIL-1β in non-infected and MG-infected house finches using the developed ELISA system. Plasma samples were collected from two individual birds from non-infected and MG-infected groups. Then, plasma samples were serially diluted with PBS as follows: 1:10, 1:20, 1:40, 1:80, and 1:160 (**a**). The diluted samples were coated with the dehydration method (60°C for 2 h), followed by sequential incubation with anti-ChIL-1β antibody (1:1000) as the primary antibody and the secondary antibody goat anti-rabbit antibody (1:2000). The HRP signal was developed with TMB solution for 30 min. The concentration of HfIL-1β plasma level was calculated using Excel (Microsoft Corp) (**b**). The values represent the mean of triplicate wells with standard deviation bars. The dashed line indicates the threshold line, representing the value of negative control and limitation of the developed ELISA system (OD450 = 0.042)

We also measured HfIL-1β plasma levels in non-infected and MG-infected birds with a 1:20 dilution factor (Fig. 4a), followed by coating onto Nunc MaxSorp® flat-bottom plates using the dehydration method. Two non-infected birds revealed 2.54 ng/mL and 3.23 ng/mL plasma HfIL-1β, whereas MG-infected house finches had 19.20 ng/mL and 8.67 ng/mL HfIL-1β levels (Fig. 4b).

## Discussion

Unlike mammalian research, few immune reagents including specific antibodies are available for immunological studies in wild birds. Although the infection of house finches by the bacterial pathogen MG is among the best-studied wildlife disease systems [22], there is still limited knowledge of the host’s immune response especially with respect to cytokine levels. Recently, our lab identified and characterized HfIL-1β, which modulates the expression of Th1/Th2 cytokines as well as enhances the expression of acute phase protein by activated immune cells [27]. We also reported increased plasma levels of HfIL-1β in MG-infected house finches using the immunoblotting method [27], which could not quantify circulating HfIL-1β levels. In this study, we developed and validated a direct ELISA system using commercially available anti-ChIL-1β antibody to quantify plasma levels of HfIL-1β. The immunoblotting assays validated the cross-reactivity of commercial anti-ChIL-1β pAb with the recombinant form and plasma HfIL-1β. Similar to previously reported (27), MG-infected house finches showed two prominent bands observed at approximately 25 kDa and 60 kDa, whereas non-infected birds showed very weak 60 kDa protein band [27], implying the 25 kDa may be the more active secreted form of HfIL-1β.

Coating, which is the process where a suitably diluted antigen or antibody is incubated until adsorbed to the surface of the well, is the first step in any ELISA. Adsorption occurs passively as the result of hydrophobic interactions between the amino acid side chains on the antibody or antigen used for coating, and the plastic surface. It is dependent upon time, temperature and the pH of the coating buffer, as well as the concentration of the coating agent. Bicarbonate buffer (pH 9.6) is the most common coating buffer, and typical coating condition involves 50–100 μL of coating buffer containing 1–10 μg/mL of either antigen or antibody incubated overnight at 4°C or for 1–3 h at 37°C. However, optimal coating conditions should be tested during the development of a new ELISA system. In this study, the typical coating condition of incubation at 4°C overnight did not work in detecting HfIL-1β in plasma samples. To increase adsorption to the plate, we applied a dehydration method for antigen coating, although we were concerned with the background level of spectrometric readings. To eliminate such background, we used the coating buffer itself as a negative control. We also incubated rHfIL-1β and random plasma samples with goat anti-rabbit antibody alone. In both cases, the absorbance at 450 nm was in the 0.034–0.044 range, which was significantly low compared to the 2 ng/mL rHfIL-1β, the minimum concentration that the established ELISA system can detect. It is currently unclear why the dehydration method rather than a typical coating method is superior for detecting HfIL-1β in house finch plasma. One potential explanation is the denaturation of HfIL-1β during the heat-based dehydration, resulting in the increased binding affinity between HfIL-1β and rabbit anti-ChIL-1β, as well as adsorption to the plate.

Unlike commercial systems, which mostly use a sandwich ELISA with capture and detection antibodies, we developed a direct ELISA by coating the antigens on the plate using dehydration. Therefore, the sensitivity of the developed HfIL-1β ELISA system is low, and the assay can only detect a limited concentration range (2–20 ng/mL). In our trials with random plasma samples and non-infected and MG-infected plasma samples, a 1:20 dilution with PBS fell within the measurable standard curve range. However, it is necessary to serially dilute plasma samples for every ELISA assay to obtain the best fit within the standard curve range. For the standard curve with rHfIL-1β, first we attempted to use a simple logarithmic equation (i.e. Y = a*ln (x) + c); however, the adjusted R-square value was 0.89, resulting in high variation among samples. Thus, we modified the logarithmic equation to “Y = a – b*ln (x + c)”, which yielded an adjusted R-square value of 0.94–0.95.

The ELISA results showed the non-infected house finches had an average of 2.89 ng/mL of plasma HfIL-1β, while the MG-infected birds had on average 13.93 ng/mL of plasma HfIL-1β. Two-sample *t*-test showed no statistical significance between non-infected and MG-infected birds (*P* = 0.28), likely due to the low sample size tested. However, the MG-infected birds showed average plasma HfIL-1β levels almost 5-fold higher than those of non-infected birds. Further studies should use a larger number of birds to examine how plasma levels of HfIL-1β change during MG infection.

## Conclusions

We validated the cross-reactivity of anti-ChIL-1β pAb with HfIL-1β using immunoblotting. Then, a direct ELISA system was developed using anti-ChIL-1β antibody to quantify HfIL-1β plasma levels using the dehydration coating method. The developed ELISA system can quantify HfIL-1β in plasma of test birds including non-infected and MG-infected house finches. This developed direct ELISA system using commercially available chicken antibody will provide valuable research and diagnostic tools for house finch research.
